# Effects of Different Exercise Strategies and Intensities on Memory Performance and Neurogenesis

**DOI:** 10.3389/fnbeh.2017.00047

**Published:** 2017-03-16

**Authors:** Kai Diederich, Anna Bastl, Heike Wersching, Anja Teuber, Jan-Kolja Strecker, Antje Schmidt, Jens Minnerup, Wolf-Rüdiger Schäbitz

**Affiliations:** ^1^Department of Neurology, University of MünsterMünster, Germany; ^2^Department of Anesthesiology, Intensive Care, and Pain Medicine, University of MünsterMünster, Germany; ^3^Institute of Epidemiology and Social Medicine, University of MünsterMünster, Germany; ^4^Department of Neurology, Evangelisches Krankenhaus BielefeldBielefeld, Germany

**Keywords:** exercise, hippocampal neurogenesis, learning and memory

## Abstract

It is well established that physical exercise affects both hippocampal neurogenesis and memory functions. Until now, distinctive effects of controlled and voluntary training (VT) on behavior and neurogenesis as well as interactions between exercise intensity, neurogenesis and memory performance are still elusive. The present study tested the impact of moderate controlled and VT on memory formation and hippocampal neurogenesis and evaluated interactions between exercise performance, learning efficiency and proliferation of progenitor cells in the hippocampus. Our data show that both controlled and VT augmented spatial learning and promoted hippocampal neurogenesis. Regression analysis revealed a significant linear increase of the amount of new hippocampal neurons with increased exercise intensity. Regression analysis of exercise performance on retention memory performance revealed a quadratic, inverted u-shaped relationship between exercise performance and retention of spatial memory. No association was found between the amount of newborn neurons and memory performance. Our results demonstrate that controlled training (CT), if performed with an appropriate combination of speed and duration, improves memory performance and neurogenesis. Voluntary exercise elevates neurogenesis dose dependently to high levels. Best cognitive improvement was achieved with moderate exercise performance.

## Introduction

An active lifestyle contributes to physical and mental health. Over the last decades, several studies have shown that the risk of contracting cardiovascular and metabolic diseases is alleviated by physical exercise (Powell and Blair, 1994). However, hard evidence for a functional relationship between exercise and brain function has been established only recently in animals as well as in humans (Hillman et al., 2008). Physical exercise improves spatial learning in healthy subjects (Colcombe and Kramer, 2003; Winter et al., 2007; Hillman et al., 2008) and conveys a protective effect against cognitive decline in aging (Wirth et al., 2014; Ngandu et al., 2015) as well as in Alzheimer’s disease (Vemuri et al., 2012) and dementia (Rovio et al., 2005; Nyberg et al., 2014; Tolppanen et al., 2015). Animal studies paralleled the results of exercise on the behavioral level (van Praag et al., 1999, 2005; Nichol et al., 2009; Yuede et al., 2009) and shed light on the underlying cellular mechanisms through which exercise influences brain function. Exercise amplifies synaptic plasticity, spine density as well as angiogenesis (Voss et al., 2013). Furthermore, physical exercise induces a robust increase in neurogenesis in the hippocampus, a brain area known to be vital for learning and also a neurogenic niche (van Praag et al., 1999; Biedermann et al., 2016).

In humans, physical exercise can be voluntary as part of a healthy lifestyle or controlled during therapeutic programs. Experimental studies reflect the different training strategies and utilize freely accessible running wheels within the animals’ home cage for voluntary exercise and treadmills and motorized running wheels for controlled exercise. Though both training regimen individually improve learning and memory and induce neurogenesis, there are notable differences between these forms of treatment (Leasure and Jones, 2008). Controlled and voluntary exercise distinctively affect growth factor expression (Ploughman et al., 2005), inflammation (Allen et al., 2015) and open-field behavior (Burghardt et al., 2004). Furthermore, controlled training (CT) has been shown to be more effective for functional and structural recovery following brain injury (Hayes et al., 2008; Schmidt et al., 2014; Schneider et al., 2014).

To investigate distinctive effects of different training strategies on memory formation and neurogenesis, we subjected mice to voluntary and controlled exercise and assessed spatial learning and memory function in the Morris water maze. Neurogenesis was ascertained by quantification of newly formed progenitor cells in the dentate gyrus. To determine direct interactions between training, brain function and neurogenesis we interrelated the exercise performance, learning efficiency and the proliferation of progenitor cells in the hippocampus.

## Materials and Methods

### Ethics Statement

All experiments were conducted in accordance with animal welfare regulations, and experimental protocols were approved by the local ethics committee. All experiments were done in a fully randomized and blinded fashion. This study was carried out in accordance with the recommendations of ethics committee of the University of Münster. The protocol was approved by the authorities of the Federal State of North Rhine-Westphalia, Germany.

### Animals

A total of 57 male C57BL/6 mice (Charles River, Sulzfeld, Germany), aged 8 weeks upon arrival were used in the experiments. They were housed individually in a 12-h reverse light/dark cycle, with lights on at 8 p.m. and kept under controlled environmental conditions (ambient temperature 22°C). Standard laboratory chow (Altromin 1324, Lage, Germany) and tap water were allowed *ad libitum*.

### Experimental Design

**Figure 1:**
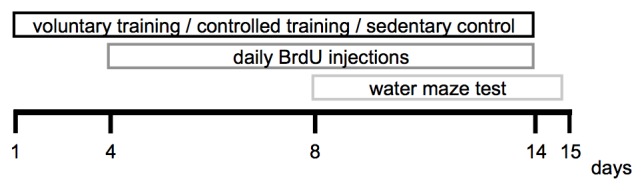
**Experimental design**.

The experimental period lasted 15 days in total. Voluntary and CT was performed between day 1 and day 14, the water maze test was conducted between day 8 until day 15. The brain tissue was extracted and processed for immunohistological analyses on day 15. The experimental design is depicted in Figure 1.

### Voluntary Training

Twenty-five animals were randomly allocated to the voluntary training (VT) group (Figure 1). The animals were housed individually in home cages equipped with running wheels (wheel diameter: 11.5 cm). The distance moved was recorded by a tachometer. Animals were habituated to the running wheels for 7 days prior to the start of the intervention. Animals that did not run a minimum of 200 m throughout that habituation period were excluded from the experiment. Five animals did not reach this criteria and were consequently excluded, a total of 20 animals remained in the VT group. During the intervention, the distance moved was documented daily.

### Controlled Training

Twenty animals were randomly assigned to the CT group (Figure 1). CT was performed in motorized running wheels (Model 80800A, Lafayette Instrument, Lafayette, IN, USA). The training was carried out during the animal’s active period (dark phase) between 9 a.m and 10 a.m. and 4 p.m and 5 p.m.

This forced exercise protocol started with a 1-week habituation, in which the animals were trained for half an hour a day and speed set to 3 m/min on day 1. The speed was increased gradually to 8 m/min until day 6. Subsequently, the training was continued twice daily with speed set to 6.5 m/min for the first 10 min and 8 m/min for the remaining 50 min of the 60-min training session. Animals run a total distance of 465 m per session (930 m per day) and 13.02 km over the 14-day training period.

### Control

Twelve animals were randomly allocated to the sedentary control group (SED) and remained in their home cages during the intervention period (Figure 1). The animals were handled daily by the experimenter.

### BrdU Labeling

For the purpose of labeling dividing cells each animal received a daily bromodeoxyuridine (BrdU) injection (50 mg/kg/d, i.p.) starting on day 4 of the intervention period and lasting until day 14 (Figure 1).

### Water Maze Testing

A Morris water maze task was used to assess spatial memory performance. The pool had a diameter of 1.50 m and was filled with opaque water; the water temperature was maintained at 21°C. The test was performed on days 8 until day 15. During the acquisition (day 8 until day 14), animals learned to use spatial cues to find a hidden escape platform located at a fixed position below the water surface. Mice were released into the pool from randomly varying positions for a maximum trial duration of 90 s. If the platform was not located, the mouse was gently guided to the platform and allowed to re-orient to the spatial cues for 10 s before being removed from the pool. On each of the acquisition days each animal performed three trials, with an intertrial interval of 60 s. During the acquisition trials, the path length to reach the platform and the swimming speed were recorded using Ethovision XT tracking software (Noldus, Wageningen, Netherlands). In the probe trial, which was performed without an escape platform on day 15, the time the animals navigated through the maze quadrants and the amount of platform crossings were recorded.

### Tissue Processing

On day 15 following the probe trial of the water maze test, animals were deeply anesthetized using a mixture of ketamine (20.38 mg/mL) and xylazine (5.38 mg/mL). Transcardiac perfusion was performed with 0.9% NaCl solution followed by 4% paraformaldehyde. The brain was removed and postfixed in 4% paraformaldehyde solution for 4 days at 4°C. The tissue was then cryoprotected by 24 h immersion in 30% sucrose-PBS solution. 40-μm coronal sections were cut with a cryostat (Leica CM 3050, Germany).

### Immunohistostaining

Immunohistological analyses were performed on brain tissue of 34 animals (SED: *n* = 8, VT: *n* = 15, CT: *n* = 11). Free-floating sections were treated with 0.6% H_2_O_2_ in Tris-buffered saline (TBS; 0.15 m NaCl, 0.1 m Tris-HCl, pH 7.5) for 30 min. Following extensive washes in TBS, sections were blocked with a solution containing TBS, 0.1% Triton-X100 and 3% normal donkey serum solution for 30 min. The same solution was used during the incubation with antibodies. Primary antibodies were applied overnight at 4°C. For epifluorescence immunodetection, sections were washed extensively and incubated with fluorochrome-conjugated species-specific secondary antibodies. The sections were placed on Superfrost Plus slides (Menzel-Gläser, Braunschweig, Germany) and mounted in Prolong Antifade kit (Molecular Probes).

The following antibodies were used: rat anti-BrdU (1:500, Accurrate), mouse anti-NeuN (1:500, Chemicon), goat anti-rat 594 (1:500, Invitrogen), goat anti-mouse 488 (1:200, Invitrogen).

### Counting Procedures

Quantification of progenitor cells was performed as described previously (Diederich et al., 2009). In short, every 6th section (240-μm intervals) of one cerebral hemisphere was selected from each animal and processed for immunohistochemistry. All BrdU-positive cells in the granule cell layer of the hippocampal dentate gyrus were counted on 10 sections per animal using a Nikon Eclipse 80i microscope. For co-labeling with neuronal marker NeuN to estimate the percentage of neurons among the newly generated cells, 50 randomly selected BrdU-positive cells per animal were analyzed. Multiplying the total number of BrdU-positive cells with the percentage of NeuN/BrdU double-positive cells yielded the number of new neurons in the dentate gyrus.

### Statistical Analysis

Randomization was carried out by the computer software “Research Randomizer” (Version 3.0; Urbaniak GC, Plous S, 2011^1^). The values presented in this study are means ± SEM. Statistical analyses were performed using the Statistical Package of Social Sciences (Version 21; SPSS Inc., Chicago, IL, USA). The normality distribution of the data was assessed by graphical examination of the histograms and verified by the Shapiro-Wilk test (*p* > 0.05). One-way analysis of variance (ANOVA) was used to compare data between groups. ANOVA with repeated measures was used when data of different groups were repeatedly collected over time. *Post hoc* comparisons were made using Fisher’s protected least significant difference (LSD) test. Associations between exercise performance, memory performance and neurogenesis were assessed by linear (polynomial) regression analysis including a quadratic term. All tests performed were two-tailed and a value of *p* < 0.05 was considered to represent a significant difference.

## Results

### Run Performance

The animals of the VT group ran an average of 3.11 ± 0.17 SEM kilometers per day. In total, they covered an average of 41.17 ± 7.17 SEM kilometers over the 14-day training period. The average range of voluntary running varied from 2.27 km to more than 100 km (100.51 km). This wide range of voluntary running allowed the use of regression analyses to ascertain the effect of individual running performance on memory performance and neurogenesis. Animals of the CT group ran 930 m per day and a total of 13.01 km during the 14-day training phase.

### Water Maze Acquisition

**Figure 2:**
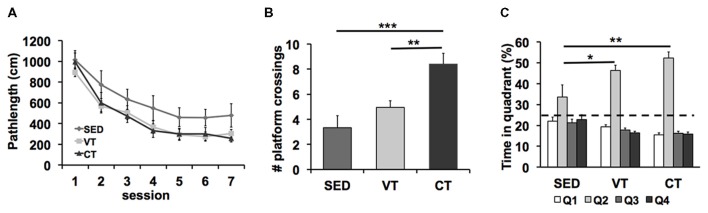
**Effects of physical exercise on spatial learning and memory formation in the Morris water maze task. (A)** Both controlled (CT) and voluntary training (VT) improved acquisition of spatial memory compared to sedentary control (SED) (SED vs. VT *p* = 0.023, SED vs. CT *p* = 0.029, Fisher’s least significant difference (LSD) test after significant ANOVA). **(B)** Analyses of the amount of crossings over the former platform area revealed a significant group effect (*p* < 0.001, ANOVA) and a significant effect of CT vs. SED (***p < 0.001, Fisher’s LSD test), no significant difference between VT vs. SED (*p* = 0.179, Fisher’s LSD test) and a significant difference between CT and VT (**p = 0.002, Fisher’s LSD test). **(C)** Analysis of the time spent in the maze quadrants showed that mice of all groups spend significantly more time in the target quadrant (Q2) than any of the other quadrants (SED: *p* = 0.041, VT: *p* < 0.001, CT: *p* < 0.001; one-way ANOVA). The target quadrant occupancy of trained mice was significantly higher when compared with SED mice (SED vs. VT: *p* = 0.02, SED vs. CT: *p* = 0.001, Fisher’s LSD test after significant ANOVA, *p < 0.05, **p < 0.01).

Analyses of the path length revealed shorter search paths for trained mice than for sedentary mice (SED vs. VT *p* = 0.023, SED vs. CT *p* = 0.029, Fisher’s LSD *post hoc* test after significant ANOVA (*p* = 0.037, *F*_(2,49)_ = 3.265), Figure 2A). No differences between the training groups were detected (CT vs. VT *p* = 0.917, Fisher’s LSD test). No significant effect of training on the swimming speed was found during the spatial learning trials (*p* = 0.247, ANOVA, data not shown).

### Water Maze Retention

A probe trial, during which the platform was removed from the pool, was performed on the eighth day of the water maze test period (Figures 2B,C).

The ANOVA revealed significant differences between the two exercise groups and the sedentary group in the amount of crossings over the former platform area (*p* < 0.001, *F*_(2,49)_ = 10.537, one-way ANOVA; Figure 2B). *Post hoc* analysis showed significant effects of CT vs. SED (*p* < 0.001, Fisher’s LSD test), no significant difference between VT vs. SED (*p* = 0.179, Fisher’s LSD test) and a significant difference between CT and VT (*p* = 0.002, Fisher’s LSD test).

Analyses of the time mice navigated through the water maze quadrants demonstrated that mice of all groups spent significantly more time in the target quadrant than in the other quadrants (SED: *p* = 0.041, *F*_(3,44)_ = 2.989; VT: *p* < 0.001, *F*_(3,76)_ = 79.721; CT: *p* < 0.001, *F*_(3,76)_ = 124.181; one-way ANOVA; Figure 2C), indicating that retention memory was detectable in all groups. The percentage of time that the trained mice spent in the target quadrant was significantly higher when compared with SED mice (*p* = 0.004, *F*_(2,49)_ = 6.265, one-way ANOVA). *Post hoc* analysis showed significant effects of VT vs. SED (*p* = 0.02, Fisher’s LSD test) and CT vs. SED (*p* = 0.001, Fisher’s LSD test) and no effect of VT vs. CT (*p* = 0.194, Fisher’s LSD test).

### Quantification of Proliferating Cells and Newborn Neurons

**Figure 3:**
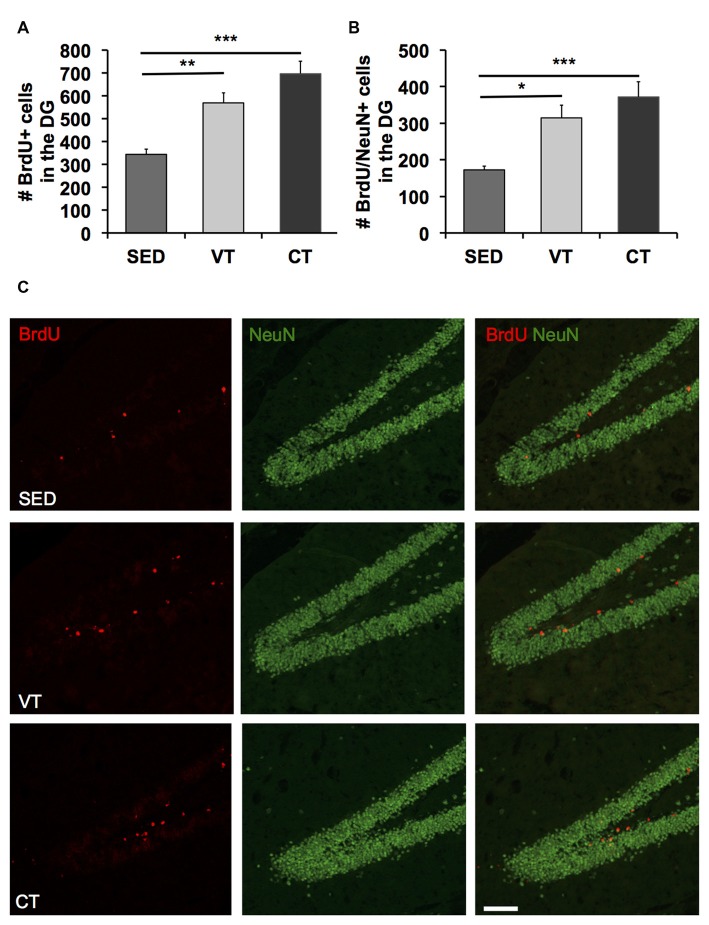
**Quantification of neurogenesis by the detection of BrdU/NeuN-expressing cells. (A,B)** Analysis of the number of BrdU and BrdU/NeuN positive cells revealed increased amounts of newborn cells in the dentate gyrus following CT and VT compared to the SED group (*p* < 0.001, ANOVA; *p < 0.05, **p < 0.01, ***p < 0.001; Fisher’s LSD test). The most prominent difference in the amount of newborn neurons was found between the CT and SED group (*p* < 0.001, Fisher’s LSD test). There was a discernible albeit insignificant difference between CT and VT group (*p* = 0.084, Fisher’s LSD test). **(C)** Photomicrographs showing representative images of bromodeoxyuridine (BrdU, red) and NeuN (green) staining in the dentate gyrus of animals of the SED, the VT and the CT group. Scale bar corresponds to 100 μm.

A one way ANOVA revealed significant differences between the three groups in the number of BrdU (*p* < 0.001, *F*_(2,31)_ = 11.510, Figure 3A) and BrdU/NeuN positive cells (*p* < 0.001, *F*_(2,31)_ = 7.988, Figure 3B). *Post hoc* analyses showed a significant increase in the amount of newborn neurons in the dentate gyrus of both training groups compared to the sedentary control group (Figures 3B,C). However, the most profound difference was found between CT and the SED group (*p* < 0.001, Fisher’s LSD test). Furthermore, there was an apparent albeit insignificant difference between CT and VT (*p* = 0.084, Fisher’s LSD test).

To estimate the number of newly generated neurons within the dentate gyrus, the number of cells-positive for both BrdU and NeuN was divided by the cells-positive for BrdU times 100. Physical training exerted no significant neuron-specific effects, as the percentage of neuronal differentiation did not differ between the groups (mean SED: 51.25 ± 3.33 SEM, mean VT: 51.73 ± 2.11 SEM, mean CT: 53.18 ± 3.56 SEM; *p* < 0.05; one way ANOVA).

### Regression Analysis of Exercise Performance on Neurogenesis

**Figure 4:**
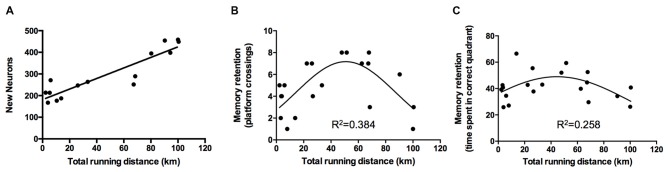
**Regression analyses of exercise performance on neurogenesis and memory retention. (A)** Analyses revealed a linear relationship between the total running distance and the amount of newborn neurons (*p* < 0.0001, *β* = 0.913). **(B,C)** Regression analyses of exercise performance on retention memory showed a quadratic, inverted u-shaped relationship for the amount of platform crossings (exercise performance: *β* = 2.194, *p* = 0.006; exercise performance^2^: *β* = −2.209, *p* = 0.006) and time spent in the correct quadrant (exercise performance: *β* = 1.659, *p* = 0.045; exercise performance^2^: *β* = −1.801, *p* = 0.031).

The exercise performance of the animals of the VT group was linearly associated with the amount of newborn neurons detected in the gyrus dentatus (BrdU/NeuN-positive cells; standardized regression coefficient *β* = 0.913, *p* < 0.0001; Figure 4A).

The difference between performance on day 1 of the acquisition and day 7 was used to assess the slope of the learning curve. Linear regression analysis did not reveal an association between the exercise performance of VT animals and the acquisition of spatial memory (slope path: *β* = −0.219, *p* = 0.3529) but revealed a quadratic inverted u-shaped relationship of exercise performance on retention memory performance as ascertained by the amount of platform crossings (exercise performance: *p* = 0.006, *β* = 2.194; (exercise performance)^2^: *β* = −2.209, *p* = 0.006, Figure 4B) and time spent in the correct quadrant (exercise performance: *β* = 1.659, *p* = 0.045; (exercise performance)^2^: *β* = −1.801, *p* = 0.031, Figure 4C).

Analysis of the amount of newborn neurons on memory acquisition as well as on memory retention revealed no significant association in any of the experimental groups (Table 1).

**Table 1:** **Regression analysis of Neurogenesis on memory performance (acquisition and retention)**.

		Voluntary training		Controlled training		Sedentary	
Independent variable	Dependent variable	*β*	*P*-value	*β*	*P*-value	*β*	*P*-value
New neurons	WM-Acquisition (slope)	−0.291	0.359	−0.304	0.363	0.136	0.771
New neurons	WM-Retention (PT, target quadrant)	−0.366	0.269	0.218	0.520	−0.590	0.163
New neurons	WM-Retention (PT, platform cross.)	−0.215	0.525	0.207	0.542	−0.537	0.214

## Discussion

Our data show that physical exercise, both voluntary and controlled, improves spatial learning and promotes hippocampal neurogenesis. A regression analysis of exercise performance and neurogenesis revealed a positive correlation between the intensity of VT and the amount of newly formed neurons in the dentate gyrus. We chose a duration of 14 days for the training phase as previous studies showed the peak of the pro-proliferative effect of physical training at 10–14 days and indicated that extended training might wear off acute effects of physical training on cell proliferation (Kronenberg et al., 2006; Fabel et al., 2009; Snyder et al., 2009).

### Moderate Physical Exercise Improves Learning and Memory Formation

There is accumulating evidence that physical exercise exerts positive effects on learning and memory formation. In the present study, both voluntary and controlled exercise improves the acquisition and retention of spatial memory. Our findings are in accordance with a number of previous studies that also showed beneficial effects of physical activity on learning and memory functions (Vivar et al., 2013). Exercise-induced improvement of cognitive abilities has been demonstrated in healthy young (Kempermann et al., 1997; van Praag et al., 1999) and old animals (Kempermann et al., 1998; van Praag et al., 2005). Furthermore, physical activity ameliorates cognitive deficits in animal models of different neurological and psychological diseases, such as Alzheimer’s disease (Görtz et al., 2008; Nichol et al., 2009), schizophrenia (Wolf et al., 2011), depression (Jha et al., 2011), and stroke (Luo et al., 2007). In analogy to the results of the animal studies, physical exercise has been shown to improve learning and memory performance in humans of different age (Hillman et al., 2008). Healthy, older subjects showed significantly improved learning and memory performance after participation in training programs (Colcombe and Kramer, 2003). A similar positive relationship between physical activity and learning performance was also shown in school children (Ahn and Fedewa, 2011) and young adults (Winter et al., 2007). Several epidemiological studies demonstrated convincingly, that a physical lifestyle might prevent or delay loss of cognitive function with aging (Flöel et al., 2008) or neurodegenerative disease like mild cognitive impairment (Geda et al., 2010), Alzheimer’s disease and dementia (Rovio et al., 2005; Nyberg et al., 2014). Overall, the results of these studies as well as the findings of the present study support the notion, that physical exercise positively affects cognitive functions in the healthy and diseased brain.

However, in the present study, a regression analysis did not unveil a linear relationship between exercise performance and successful memory formation. Instead the most effective memory retention was found in animals with medium running performance. In the present study, the average range of voluntary running varies from 2 km to more than 100 km over the 14-day training period. Previous studies linked excessive voluntary exercise to pathologic and maladaptive behavior (Richter et al., 2014). In mice selectively bred for high voluntary wheel running, high exercise performance increases hippocampal neurogenesis but impairs spatial learning (Rhodes et al., 2003, 2005). A recent study found that the endogenous stimulation of hippocampal neurogenesis after brain injury coincides with an increased rate of aberrantly integrated neurons and concluded, that this may contribute to functional deficits including cognitive impairment (Niv et al., 2012). Abnormal hippocampal neurogenesis is also thought to contribute to cognitive impairments in temporal lobe epilepsy (Parent and Murphy, 2008). Although not investigated here, excessive exercise might elicit increased but aberrant neurogenesis, which may explain the lack of benefit from high intensity exercise. In humans, accumulating evidence also suggest that excessive exercise might not only be less beneficial but potentially harmful. Intense cardiovascular exercise has been shown to elicit generalized fatigue and depression associated with changes in the expression of neurotransmitters such as glutamine, dopamine and 5-HTP and the production of large quantities of proinflammatory cytokines such as IL-1β, IL-6 and TNF- α (Smith, 2000).

### Physical Exercise Enhances Hippocampal Neurogenesis Dose Dependently

The present study shows that controlled as well as voluntary exercise enhances hippocampal neurogenesis. This finding corroborates previous studies and confirms the notion that hippocampal neurogenesis might be an essential part of the structural mechanism underlying the exercise-induced improvement of cognitive functions. The increase in neurogenesis is one of the most reproducible effects of physical activity on the brain and physical training the strongest stimuli of hippocampal neurogenesis (van Praag, 2009). The landmark study from van Praag et al. (1999) showed enhanced hippocampal neurogenesis associated with improved acquisition and retention of spatial memory in healthy young mice following 42-day exposition to running wheels. Similar effects of exercise on cognition and neurogenesis were demonstrated in old animals (van Praag et al., 2005) and, furthermore, physical exercise ameliorated or even reversed deficits in adult neurogenesis induced by irradiation (Naylor et al., 2008) or genetic ablation (Sakalem et al., 2017).

Even though many studies reported increased neurogenesis following exposure to a running wheel, a direct correlation between running performance and proliferation of neural cells was rarely performed. In this study, we confirmed a linear relationship between running performance and neurogenesis. In line with these results, Allen et al. (2001) demonstrated a correlation between exercise performance of mice running in running wheels and the amount of cell proliferation and survival in the hippocampus. These results indicate that voluntary running promotes neurogenesis in a linear, dose-dependent manner.

### A Non-Linear Relationship between Neurogenesis and Spatial Memory Performance

Numerous studies suggested that learning and neurogenesis might be closely related (for review see Vivar et al., 2013). The present study also demonstrated that physical exercise, controlled and voluntary, improved the acquisition and retention of spatial memory and promoted hippocampal neurogenesis. However, a linear relationship between newly formed neuronal cells in the hippocampus and memory acquisition and retention could not be found. Previous studies tried to establish a functional relationship between neurogenesis and cognitive function by using animals with different neurogenic activity due to a specific genetic background or different age. Kempermann and Gage (2002) tested mice in the Morris water maze, which differed genetically in terms of hippocampal neurogenesis and learning abilities. The animals with the lowest number of new neurons showed the worst results in the acquisition of spatial memory. However, no correlation between hippocampal neurogenesis and memory performance was found in the retention of acquired spatial memory. Working with rats, studies from Merrill et al. (2003) and Van der Borght et al. (2005) found no correlative relationship between strain- and age-dependent differences in hippocampal neurogenesis and spatial learning. Together with previous studies, our results demonstrate that neurogenesis might be a crucial mechanism for effective learning and memory formation. However, while neurogenesis might be essential for learning and memory processes, more new progenitor cells do not necessarily translate into further improved memory performance. Survival, differentiation and successful integration of these newborn cells might be more important for these cells to become functional and subsequently influence behavior.

### Distinctive Effects of Controlled and Voluntary Training on Neurogenesis and Memory Performance

Most studies investigating the effects of exercise on learning and memory utilize either forced or VT, with the vast majority using VT as exercise paradigm. While behavioral and structural benefits of voluntary exercise on learning performance and neurogenesis were repeatedly confirmed, forced training elicited inconsistent results. These inconsistent results may be attributable to different training modalities as these often differ in terms of duration and intensity. Mild to moderate training predominantly elicits a beneficial effect on learning performance and neurogenesis, whereas intensive training can even have a negative impact (Kim et al., 2003; Albeck et al., 2006; Lou et al., 2008). An important role in this context seems to be the presence of stress as it has been shown to impair memory performance (Sandi and Pinelo-Nava, 2007) and reduce neurogenesis (Schoenfeld and Gould, 2012). Intensive forced exercise does elevate corticosterone levels to a much greater extent than voluntary (Ploughman et al., 2005) and moderate CT (Inoue et al., 2015). Moderate training does not increase stress responses above those elicited by VT (Leasure and Jones, 2008) and a recently published study using a moderate controlled exercise paradigm similar to this used in the present study found no signs of emotional or physical disturbance following the training intervention (Sakalem et al., 2017). Taken together, previous studies together with the findings presented here suggest, that controlled exercise promote memory function and increases neurogenesis when applied moderately without inducing potentially detrimental stress responses.

In the present study, controlled exercisers only covered less than one third of the average distance run by voluntary exercisers every day, but achieved similar improvements of memory performance and neurogenesis. Regularity of controlled exercise may be one explanation for the high efficacy of CT. A study by Li et al. (2013) showed that regular controlled exercise, performed daily at the same time-of-day, speed and duration, increases neurogenesis, improves spatial memory performance and decreases the level of corticosterone compared to irregular exercise performed either at the same time-of-day and speed, but for randomly varying duration or at the same speed and duration, but randomly varying time-of-day. Furthermore, a CT program prevents excessive running, which is regarded as a maladaptive behavior (Richter et al., 2014) and might lead to impaired memory performance.

## Conclusion

Different forms and intensities of physical exercise affect the structure and function of the brain differently. Here, we show that moderate controlled and voluntary exercise improves neurogenesis and memory performance. Voluntary exercise elevates neurogenesis dose dependently to high levels and best cognitive improvement was elicited by moderate exercise performance. Further studies have to focus on the cellular mechanisms underlying the distinctive effects of physical exercise on neurogenesis and memory functions.

## Author Contributions

KD, JM, J-KS, AS and W-RS conceived the study and coordinated the experiments. AB and KD performed the experiments. HW, AT and KD analyzed the data. KD wrote the manuscript. All authors read and approved the final manuscript.

## Conflict of Interest Statement

The authors declare that the research was conducted in the absence of any commercial or financial relationships that could be construed as a potential conflict of interest.

## Footnotes

^1^www.randomizer.org/
